# Immersive Experience and Climate Change Monitoring in Digital Landscapes: Evidence from Somatosensory Sense and Comfort

**DOI:** 10.3390/ijerph20043332

**Published:** 2023-02-14

**Authors:** Zhengsong Lin, Ziqian Yang, Xinyue Ye

**Affiliations:** 1 Virtual Landscape Design Lab, School of Art and Design, Wuhan Institute of Technology, Wuhan 430205, China; lzs2020@wit.edu.cn (Z.L.); 22112020024@stu.wit.edu.cn (Z.Y.); 2 Department of Landscape Architecture and Urban Planning, Center for Geospatial Sciences, Applications and Technology, TAMIDS Design and Analytics Lab for Urban Artificial Intelligence, Texas A&M University, College Station, TX 77840, USA

**Keywords:** digital landscape, emotional preference, somatosensor comfort, ancient tree ecological area

## Abstract

In this study, the virtual engine software (Unity 2019, Unity Software Inc., San Francisco, California, the U.S.) was used to generate a digital landscape model, forming a virtual immersive environment. Through field investigation and emotional preference experiments, the ancient tree ecological area and the sunlight-exposed area were respectively monitored, and the somatosensory comfort evaluation model was established. The subjects showed the highest degree of interest in the ancient tree ecological area after landscape roaming experience, and the mean variance in SC fluctuation was 13.23% in experiments. The subjects were in a low arousal state and had a significant degree of interest in the digital landscape roaming scene, and there was a significant correlation between positive emotion, somatosensory comfort and the Rating of Perceived Exertion index; moreover, the somatosensory comfort of the ancient tree ecological area was higher than that of the sunlight-exposed area. Meanwhile, it was found that somatosensory comfort level can effectively distinguish the comfort level between the ancient tree ecological area and the sunlight-exposed area, which provides an important basis for monitoring extreme heat. This study concludes that, in terms of the goal of harmonious coexistence between human and nature, the evaluation model of somatosensory comfort can contribute to reducing people’s adverse views on extreme weather conditions.

## 1. Introduction

In parallel with economic development, global warming and extreme weather events occur frequently, causing harm to the environment, production systems, and human health. The rapid economic growth in China has intensified the consumption of fossil fuels such as coal and oil for power generation and transportation, which leads to environmental degradation and extreme weather events through the emission of carbon, sulfur and nitrogen [1]. Studies in Pakistan have found that the adverse impact of climate change on wheat crop damage increases with the more frequent extreme climate, especially closer to the harvesting period [2]. In addition, excessive energy inputs cause extreme weather, so hybrid optimization techniques have been used to predict CO_2_ equivalent emissions from fisheries [3]. How to create a comfortable living environment in the context of climate change has become an urgent issue.

## 2. Literature Review

In recent years, in order to cope with extreme global climate change, academics have put forward a series of countermeasures that have been inspired by the Paris Climate Change Conference and the United Nations Biodiversity Summit, but the effect is not obvious [4,5]. This failed experience prompted people to change their ways. Xu (2018), Chang (2015) and Yu (2017) believe that the idea of “unity of nature and man” is the basis of contemporary ecological construction, and that the cultural heritage protection of traditional Chinese villages should apply the philosophy of “unity of nature and man” [6,7,8]. Singh et al. (2020) and Chadalavada et al. (2021) believe that environmental change will affect grain yield, and Xu (2022) strives to capture and store CO_2_ on a large scale through a BECCS model [9,10,11]. Scholars have extracted ancient ecological protection theories from the landscape patterns of traditional villages, and some authors have used ancient trees in cultural heritage projects as an intermediary to explore theoretical methods of environmental protection. Lindenmayer et al. (2017) and Depardieu et al. (2020) believe that the protection of ancient and famous trees is conducive to seeking historical evidence of ancient people’s pursuit of ecological views and of establishing a symbiotic order between human being and human being, human being and nature, and human being and society, through the good ecological environment around ancient trees [12,13]. In a study on extreme climate management, Ni (2020) believes that digital technology opens a new chapter for landscape design and expression [14]. The digital visualization of extreme climate governance is to transform information into graphics and to give people the most intuitive effect. Technologies such as Augmented Reality (AR), Virtual Reality (VR) and Mixed Reality (MR) have emerged gradually since 2016. Li et al. (2022), Zhao et al. (2021) and Shan et al. (2021) put forward the concept of “digital landscape”, believing that humans’ direct senses express the communication process between human and nature. Software such as virtools, Cult3D and Quest3D can be used to experience human feeling and body [15,16,17]. Aoki et al. (2017) advocates the use of digital restoration AR technology to promote the development of cultural and creative economy, and the dissemination of tourism and cultural features [18].

The Embodied cognition theory refers to the strong relationship between physical experience and mental state, as a physical experience-activated mental experience [19]. Denmark et al. (2020) argue that with the wide application of various modern information technologies, such as VR and AR, in environmental construction, the principle of immersive reality has become a prospective issue that needs further research. By analyzing the LEAF model, a more robust explanation is provided as to how the body plays a role in collaborative learning [20]. Tactile imagery refers to a mental process similar to imagination that can be generated by text description, picture description, mental cue words or other external stimuli; this is for things that an individual has not yet experienced, and is called mental imagery [21]. Fei et al. (2020) analyze consumers’ preference for software icons through tactile imagery and believe that tactile imagery plays an important role in marketing [22]. Emotional arousal causes the body and mind to respond to external stimuli. Anderson et al. (1995) improve the subjective evaluation questionnaire by using discomfort instead of comfort as the evaluation method, establishing the questionnaire of seat discomfort, and comprehensively summarizing the factors affecting comfort [23,24]. The fatigue index was also evaluated by the non-interventional monitoring of physiological parameters. Wang et al. (2022) propose that detection methods such as electrodermal activity (EDA), heart rate (HR) and heart rate variability (HRV) could be used to effectively monitor human fatigue [25]. The research on somatosensory comfort level can optimize the evaluation of comfort through a variety of objective physiological data and subjective feeling evaluation. Zhang et al. (2010) argue that the somatosensory comfort level is based on the principle of heat exchange between the human body and the near-ground atmosphere, and that it plays an important fundamental role in urban ambient weather readings [26]. Heiman et al. (1982) establish the formula of “comfort level” by referring to human discomfort level, temperature and humidity level [27]. The ancient trees in the cultural heritage sites show the evolution of natural changes in climate, geography, vegetation and ecology over thousands of years. According to Rigo (2021), old and valuable trees are important resources for urban landscape and ecological construction, and play an important role in improving and maintaining the ecological environment [28].

In order to mitigate the impacts of climate change, the goal set forth by the international community is to reach a maximum point in carbon dioxide emissions before 2025 and to become carbon neutral by 2050 [29]. Since the Convention for the Protection of Intangible Cultural Heritage was adopted by the United Nations in 2003, the number of research papers on Intangible Cultural heritage at home and abroad has increased year by year [30]. Hubei Province issued the Measures for the Protection and Management of Ancient and Famous Trees in 2010 [31].

To solve the problem of global warming and extreme weather, scholars around the world have put forward many measures, but most of the theories start from the social problems and less commonly consider the human itself. It indicates that today’s global climate governance and ecological protection bodies should change their thinking, learn from the theories and methods related to the protection of traditional villages from the perspective of international heritage protection, apply heritage protection theories to further research, and actively introduce ecological principles to achieve a win–win situation between development and environmental protection. There are few studies on the protection of ancient and famous trees and the restoration of ancient ecology in cultural heritage sites. At present, the important role of ancient trees in the study of ecological environmental protection is only in the theoretical stage. In addition, ways in which to conduct compound research on ancient trees, somatosensory comfort level and the harmonious development of human and nature have not been practiced yet. As for ecological comfort experience, there are human–computer interaction experiences that use software such as Digital Landscape, Virtools and Cult3D, as well as the application of AR technology in the tourism industry, but these experiences only stay through the mouth, eyes, ears and other senses. However, research on human experience monitoring, considering factors such as muscle, skin and psychology, is still far from enough, and the technology cannot directly observe the data monitoring of the subjects. Most of the research on tactile mental image and concrete cognitive theory are based on visual and immersive teaching, but there is little research on the interaction between different senses. Research on emotional arousal is mainly based on visual stimuli. Relatively little research on emotional arousal can be experienced and detected through VR, and few applied studies have focused on people’s subjective feelings and somatosensory comfort level.

In conclusion, this study takes the frequent occurrence of global extreme weather in recent years and the lack of a substantial improvement in the global governance of ecological environment protection as the prompt, takes ancient and famous trees as the research object, and takes the restoration of the good ecological environment of cultural heritage sites as the breakthrough point to build a digital landscape model and immersive experience scenario simulation. With the support of concrete cognitive theory and tactile mental imagery, a virtual immersion experience was integrated into human psychological perception, and then embodied through physiological representation. Finally, the advantages and disadvantages of somatosensory comfort were evaluated from the perspective of enhancing human somatosensory comfort, so as to form an innovative model of “Immersive scenario simulation–somatosensory comfort evaluation–climate change monitoring”. In addition, local residents, tourists and cultural inheritors were invited as subjects to take part in the virtual immersion experience. After that, subjects were invited to fill in the questionnaire and record the data to verify the feasibility and timeliness of this innovative model by evaluating the somatosensory comfort.

## 3. Data Sources and Methods

### 3.1. Location Overview

**Figure 1:**
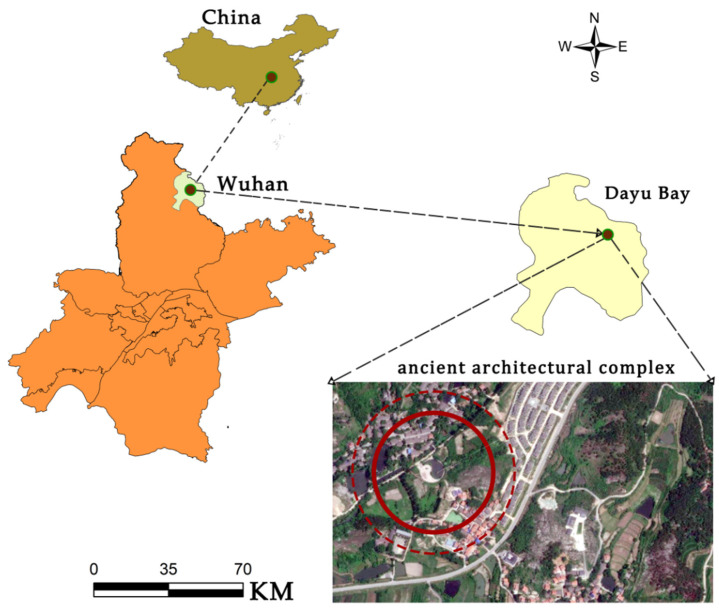
Location analysis diagram of Huangpi Dayu Bay. Source: Google Maps intercept image.

Dayu Bay is a state-level historical and cultural village in Wuhan, Hubei Province (114° E, 31° N) [32], which has a history of 600 years. All of its sites, road structures and ancient streets have unique historical and cultural features [33,34]. According to the genealogy of the Yu Family, in the second year of Hongwu of the Ming Dynasty (1369), Zhu Yuanzhang ordered a large migration to Ganhu, and the Yu family, who lived in Wuyuan and Dechuan of Jiangxi Province, then moved there [35,36]. It was identified as a “Feng Shui” treasure land by the Yu family; thus, it was named Dayu Bay [37,38,39], as shown in Figure 1.

### 3.2. Field Investigation

**Figure 2:**
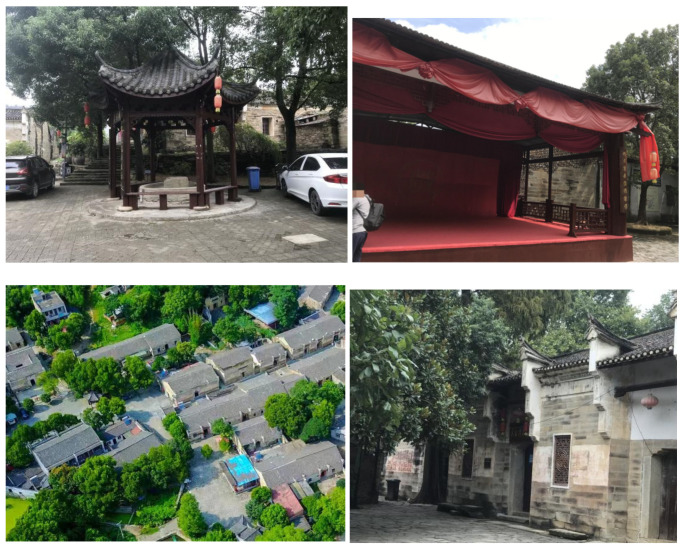
Field survey of Dayu Bay. Source: The author photographed and obtained the written consent of the traveler.

In this study, the TITAN360 panoramic camera (Lanfeng Chuangsee Network Technology Company, Shenzhen, China) and DJI Inspire Ⅱ UAV (DJI Innovation Technology Company, Shenzhen, China) were used to photograph and measure the ancestral temples, buildings and canals. The survey found that there are 47 fully preserved ancient buildings of the Ming and Qing Dynasties in Dayu Bay, but due to disrepair and a lack of protection, many buildings have been privately demolished by villagers and are in a state of dilapidation. Two small streams in this village, that is, the inlet and outlet of Stone Creek, converge in the central reservoir, and the villagers have given up using drainage channels. In this area, there is a living 410-year-old Hubei second-class ancient tree and about 20 famous ancient trees with an age of 100–200 years; these are all around the ancestral temple, as shown in Figure 2. By monitoring and comparing the temperature, humidity and wind speed around the ancient tree ecological area and the sunlight-exposed area, it was found that the somatosensory comfort level around the ancient tree ecological area was better than the other sunlight-exposed area. A total of 70 questionnaires were distributed and 64 were collected. The questionnaires included the gender, age and occupation of the subjects, and the comfort data of villagers, students and cultural inheritors of Dayu Bay on the existing body of Dayu Bay were collected.

### 3.3. Construction of Virtual Scenes

**Figure 3:**
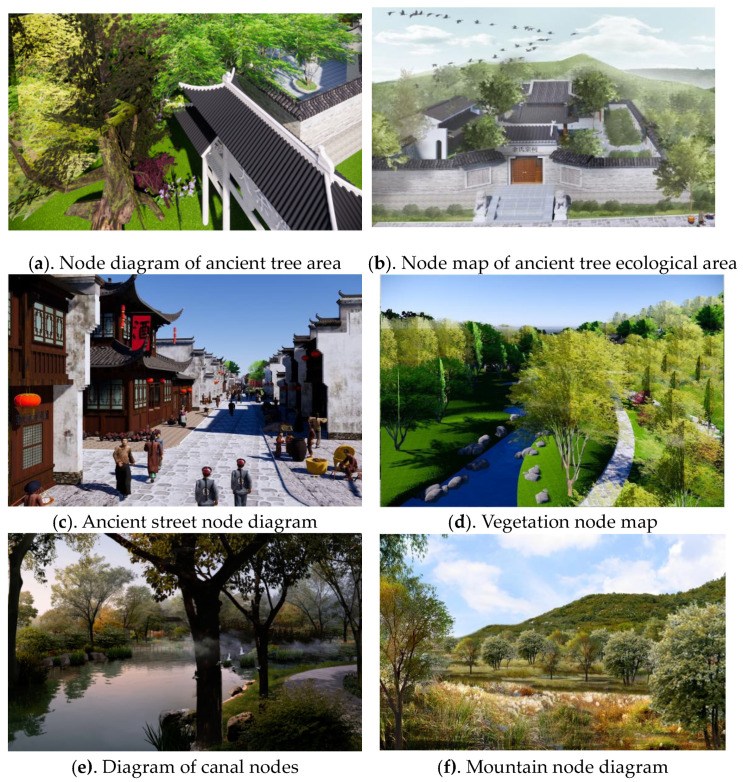
Design drawing of Dayu Bay. Source: Figures (**a**–**f**) are all virtual scene node diagrams drawn by the author.

Taking ancient trees as the research object and restoring the good ecological environment of cultural heritage sites as the starting point, this study constructs the digital landscape model and immersive experience scenario simulation. Its working principles were as follows: First, it built a digital landscape scene by using SketchUP (Google Company, California, American). Secondly, Unity was used to set a VR-Plugin program. In the restoration of ancient scenes, scripts were added to five parts, including buildings, streets, canals, plants and ancient tree ecological areas, and the collision body of the mesh collider was added. Finally, after exporting the roaming program, the ErgoLAB platform was used for the virtual immersion experience, and the experience data of the subjects were recorded, as shown in Figure 3.

### 3.4. VR Eye Movement and Skin Electrical Experiments

The behavioral experiment and field investigation were used to form a control group. The experimental group invited the subjects to experience the digital landscape scene. Through the experience data in the electrical experiments on the skin, the influence of the somatosensory comfort level in the digital landscape roaming scene on the emotion fluctuation of the subjects was monitored.

#### 3.4.1. Experimental Hypothesis

In the immersive experience of digital landscape, the emotions of subjects can be aroused through tactile mental images. The greater the interest, the lower the arousal.

The subjects’ somatosensory comfort level is related to positive emotions. The higher the somatosensory comfort level of digital landscape simulation, the higher the subjects’ satisfaction is, and the lower their emotional arousal is.

In reference to the “Huangpi County Chronicle” [40], the construction of a digital landscape roaming scene can better show the landscape ecological pattern than the contemporary scene, and can create a more scientific and reasonable condition for the analysis of the somatosensory comfort level.

#### 3.4.2. Experimental Design

In this experiment, an inter-subject design of 2 (high/low interest) × 2 (high/low emotional arousal) × 2 (high/low somatosensory comfort) was used. The independent variables were high/low interest and the high/low emotional arousal of the subjects’ roaming experience. The dependent variable was the high/low scene somatosensory comfort of the subjects in the roaming landscape experience. The comfort level of the subjects during the digital landscape roaming experience, and the eye tracking and somatosensory comfort level after the experiment were analyzed and processed; in addition, the difference in the somatosensory comfort level between the control group was compared, in order to provide a new approach to mitigating the frequent occurrence of global climate extremes.

#### 3.4.3. Experimental Materials

Selection of subjects: students, experts, residents and cultural heritage experts were selected as subjects in the experiment. The subjects were between the ages of 18 and 70 and were in good health, with normal vision and no vertigo for the 3D experiment. The questionnaire mainly collected the somatosensory comfort data of the subjects in the ancestral temple, street, canal and other areas in the digital landscape scene.

Hardware equipment: Based on the field investigation in the early stage, we used Mavic 2 version DJI UAV produced by DJI Innovation Company in Shenzhen, a range finder, a HUAWEI mobile phone produced by HUAWEI Technology Co., Ltd. in Dongguan and other equipment to sort out and summarize the survey data of terrain, buildings, streets and canals. In the experiment, VIVE pro series eye movement equipment (model V1.0 produced by HTC Electronics Co., Ltd. in Taiwan) with a sampling rate of 120 Hz, EDA skin electrical equipment, a HP laptop (LAPTOP-8M7P9IKS) and a HP desktop computer were used for digital landscape scene simulation and experimental data collection. The measuring instrument was a TES-1360A hygrograph and TES thermal anemometer; the measuring parameters were air temperature, air humidity and wind speed. The instrument was placed 1.50 m above the horizontal ground and recorded every 60 min.

Software equipment: ErgoLAB platform version 3.0 (KingFar International Inc., Beijing, China), SketchUp 2018 and Unity 2019 were used for digital landscape scene simulation and experimental data collection.

#### 3.4.4. Experimental Process

Before the experiment: Firstly, based on the field investigation, the existing conditions, buildings, vegetation and infrastructure of Dayu Bay were photographed and sorted by using drones, rangefinders and mobile phones. At the same time, questionnaires were conducted among local residents, students and cultural heritage experts to collect and sort out relevant county records and ancient trees of Dayu Bay. The subjects in this group were the control group. Meteorological statistics show that summer in Huangpi District in Wuhan has started, on average, on the 137th day of the year in the past ten years. Based on this, the measured time periods of this study were selected as days 8, 15, 22 and 29 of June 2022. The measured analysis was conducted from 9:30 to 18:30 every day, and the monitoring was conducted simultaneously in the sunlight-exposed area and the ancient tree ecological area, respectively. Then, the subjects entered the simulation scene to start the virtual eye movement experiment and EDA experiment. Next, the digital landscape roaming model of Dayu Bay was restored. Finally, the digital landscape roaming model was imported into Unity 2019 for program setup to ensure that the eye tracker could detect objects and record eye movement data.

**Figure 4:**
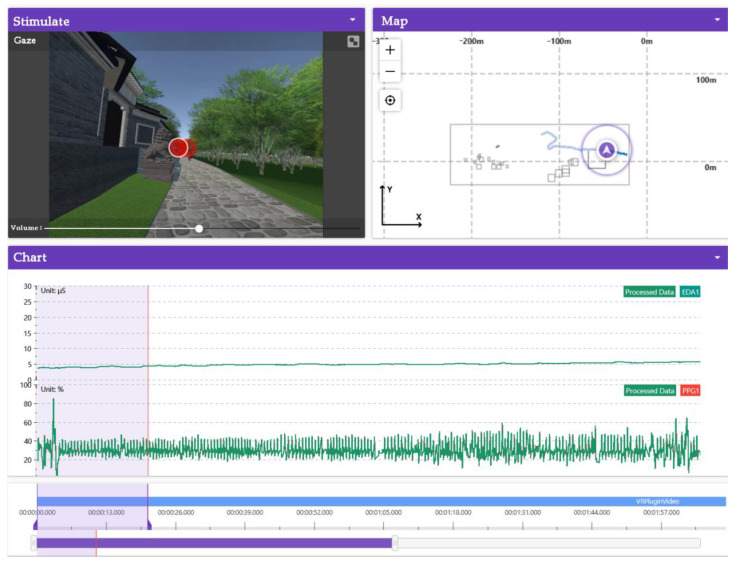
Experimental scene and data monitoring interface. Source: ErgoLAB platform exported, data detection screen after subject’s roaming experience, EDA, PPG data.

In the experiment: The subjects wore a VR headset and EDA electrical equipment on the skin, and the position of the helmet was adjusted to make the eye level line position correct; then, the program was connected with the ErgoLAB platform, and 70 subjects were invited to take part in the digital landscape scene roaming experience. It was ensured that the eye tracking and electrical data from the skin were normal and could be accurately recorded. The experiments were conducted locally in Dayu Bay from 8–10 June 2022, and were monitored in the ecological zone of ancient trees and the sunlight-exposed zone, respectively, in order to ensure a stable experimental environment and accurate data. The experimental interface is shown in Figure 4.

After the experiment: After the experiment, 70 subjects were invited to take an immersive experience questionnaire; the subjects’ interest levels in the ecological creation of different interest areas in the virtual scenes were collected, and this was used as the basis data to analyze the subjects’ physical comfort in the virtual scenes. This group of subjects was the experimental group. After removing 6 invalid samples, 64 valid samples (42 females and 22 males) were obtained from the questionnaire, and the effective sample rate was 88%. SPSS21.0 software was used to analyze the reliability and validity of the questionnaire. Cronbach’s α was 0.688, indicating that the reliability of the questionnaire was acceptable. KMO and Bartlett tests were carried out through the results of the questionnaire, showing that the KMO value was 0.631, and the *p* value of significance was 0.000, which was significant at the level. The null hypothesis was rejected, showing that the variables were correlated, and the factor analysis was effective.

#### 3.4.5. Experimental Results

1. Visual analysis of interest experience.

In this study, the reliability and validity of the two questionnaires for the control group were analyzed. The Cronbach’s α coefficient values of the questionnaire model before and after the experiment were 0.804 and 0.688, respectively, indicating that the reliability was good. The KMO values were 0.852 and 0.631, respectively. The Bartlett test showed that the *p* values of the two questionnaires were 0.000 and 0.000, respectively. This indicates that the values of the two questionnaires are significant and the variables are correlated.

**Figure 5:**
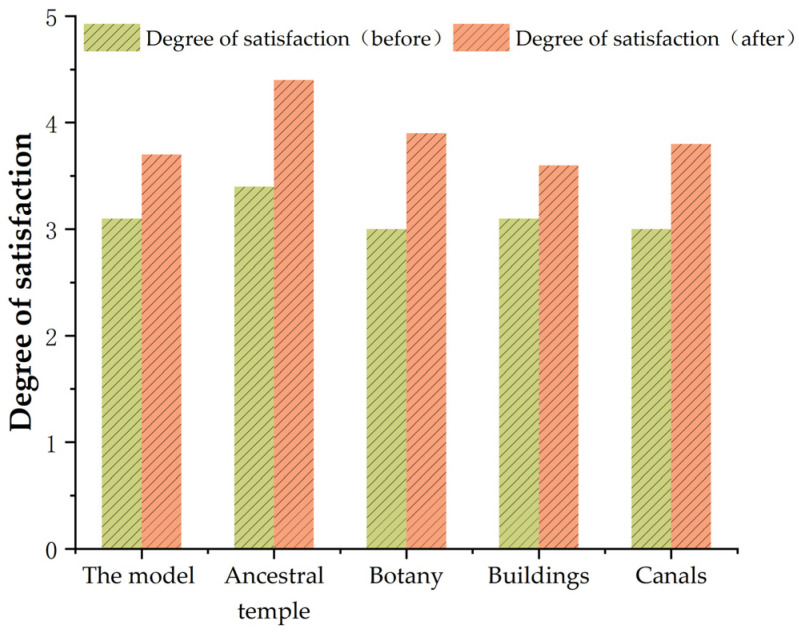
Likert scale satisfaction trend chart. Source: The average of satisfaction was obtained from the statistical data of questionnaire data, and the level of satisfaction was compared before and after the experiment.

A comparison and analysis of the questionnaire group data and experimental data showed that the somatosensory comfort of the subjects after immersion in the digital landscape scene was higher than that in the sunlight-exposed area, and the interest of the subjects was the highest in the ancient tree ecological area, as shown in Figure 5.

After 3 min of the immersion experience, in order to observe whether the area of interest (AOI) stimulates the subjects’ interest, the effectiveness of the immersion experience was measured by the level of interest. According to the research results of Constantin et al. (2021) and Natarajan et al. (2005), the degree of interest represents the interest weights of subjects in different regions of interest, and the larger the weight, the higher the interest of subjects in a scene or a certain region [41,42]. In this study, the degree of interest is calculated according to the subjects’ gaze time and pupil diameter in the digital landscape roaming immersive experience [43,44], as follows:

(1) $$Y_{ij}=\frac{\max\;x_{ij}-x_{ij}}{\max\;x_{ij}-x_{ij}};i\in \left[1,m\right],j\in \left[1,n\right]\;\mathrm{and}\;Y_{ij}=\frac{x_{ij}-\min\;x_{ij}}{\max\;x_{ij}-\min x_{ij}};i\in \left[1,m\right],j\in \left[1,n\right]$$

(2) $$f_{ij}=Y_{ij}/\sum_{j=1}^{n}Y_{ij}$$

(3) $$I_{i}=\frac{t_{i}}{\sum_{i=1}^{n}t_{ij}}+\frac{c_{i}}{\sum_{i=1}^{n}c_{ij}}+\frac{p_{i}}{\sum_{i=1}^{n}p_{ij}}$$

In the formula, *I_i_* is the ith degree of interest, *t_ij_* is the time from the *i^th^* fixation point to the *j^th^* fixation point, *c_ij_* is the time from the *i^th^* fixation point to the *j^th^* fixation point, *p_ij_* is the pupil diameter from the *i^th^* fixation point to the *j^th^* fixation point, and *n* is the total number of fixations.

2. Correlation analysis between virtual immersive experience and emotional arousal

**Figure 6:**
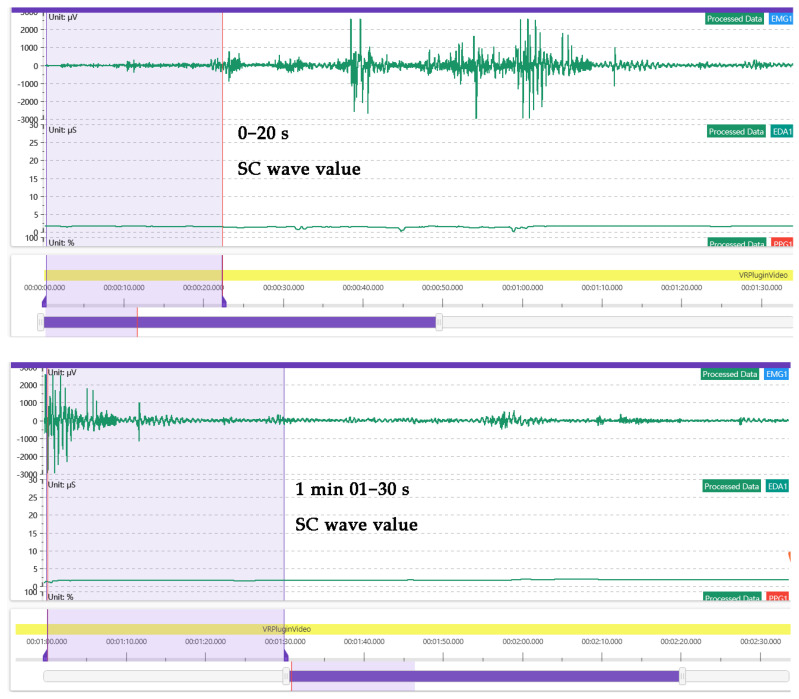
SC fluctuation value extraction. Source: ErgoLAB Platform Screenshot. The emotional arousal state during 0–20 s and 1–30 s of 1 min was intercepted to calculate the SC range of the subjects.

The correlation between the virtual immersive experiences and emotional arousal were analyzed. Based on the theory of representational cognition and tactile imaging, this study analyzes the SC wave value. According to the theory of concrete cognition, there is a strong connection between physical experience and mental state, and mental state activates physical experience [45]. The psychological changes in the subjects during the immersive experience had an effect on the skin conductance. Human skin resistance and conductivity vary with the function of the skin’s sweat glands. These measurable electrical changes in the skin are called EDA, which consist of skin conductance (SC), skin conductance level (SCL) and skin conductance response (SCR). SC is the most sensitive indicator for assessing the level of emotional arousal. The range of SC was calculated by intercepting the difference between 0–20 s and 01–30 s of 1 min after stimulation, and was used to analyze the fluctuation in SC, as shown in Figure 6. According to Ward (2003), fluctuations in SC skin conductivity tend to increase when the subject’s mood increases. When the SC wave value fluctuated more than 7%, sympathetic activity was dominant, with hyper-emotion and an increased psycho-cognitive load. When the SC wave value is less than 7%, the fluctuation in SC skin conductance shows a downward trend, the parasympathetic nerves dominate, emotional recovery is calm, and the psychological cognitive load is low [46]. SPSS software was used to analyze the correlation between interest and SC.

3. Correlation analysis between emotional arousal and somatosensory comfort level

This part was performed in two steps. Step 1: This study measured the somatosensory comfort under two scenarios: the ancient tree ecological area and sunlight-exposed area. Jin et al. (1996) and Katia et al. (2014) believe that ancient and famous trees can preserve soil and water, regulate temperature and optimize the ecological environment [47,48]. Lai et al. (2020), De et al. (2021) and Addas (2022) believe that plants can improve the local microclimate and air quality, and can affect human comfort in the ecological environment [49,50,51]. In this study, the calculation equation for the somatosensory comfort level is proposed as follows:

(4) $$I_{CHB}=1.8t-0.55(1.8t-26)(1-RH)-3.2\sqrt{V}+32$$

where *I_CHB_* is somatosensory comfort level, t is air temperature (℃), V is wind speed (m/s), and RH is relative humidity (%). According to Jin (2018), when the somatosensory comfort is in the range of 1–50, it is defined as the low temperature interval; the 75–100 range is defined as the high temperature range; the 50–80 range is defined as the comfort zone; and the 58–70 range is the best comfort interval [52]. In this study, the monitoring data of the somatosensory comfort level under the two scenarios of ancient tree ecological area and sunlight-exposed area were compared and analyzed to obtain the best effect of somatosensory comfort level under the two scenarios.

Data of *t*, *RH* and *V* were obtained from field survey. On days 8, 15, 22 and 29 of June 2022, four times within a month, data for the ancient tree ecological area and sunlight-exposed area were recorded from 9:30 to 18:30 every day: within 30 time periods, data for factors such as temperature, humidity and wind speed were obtained, and the average deviation value was between 0.20 ± 0.50. A comparative analysis of somatosensory comfort level was made between the ancient tree ecological area and the sunlight-exposed area, as shown in Table 1.

The second step was to analyze the correlation between emotional arousal and somatosensory comfort. Firstly, the fatigue degree of the subjects after the immersion experience was monitored by RPE. According to Seijo et al. (2015), the self-assessed fatigue scale (Rating of Perceived Exertion, RPE) was used to analyze the fatigue degree; this is a kind of subjective feeling that is used to calculate the load of the effective methods (in the RPE scale, 13 is the threshold. More than 13 indicates fatigue and less than 13 indicates comfort) [53]. RPE and optimal heart rate are calculated using the following formula:

(5) RPE=HR÷10

(6) BH=(220−AVA)×80%

where RPE is the fatigue degree of the subjects, and RPE × 10 is the heart rate (HR) level of the subjects. BH is the best heart rate of the subjects, AVA is the average age of the subjects 25.65 ± 10.26, and 80% is the maximum heart rate of the subjects.

According to Equations (5) and (6), when RPE ≥ 13 and the optimal heart rate is between 116.80 and 130.60 bpm, the subjects enter into a state of fatigue, with obvious negative emotions and low somatosensory comfort. When RPE < 13 and the optimal heart rate < 116.8 bpm, positive emotions were obvious and the somatosensory comfort level was high. This result is consistent with that of Crewe et al. (2008), Asadi (2014) and Corbett et al. (2009) [54,55,56]. SPSS software was used to analyze the correlation between somatosensory sense and comfort, RPE and SC.

#### 3.4.6. Logical Structure Visualization

**Figure 7:**
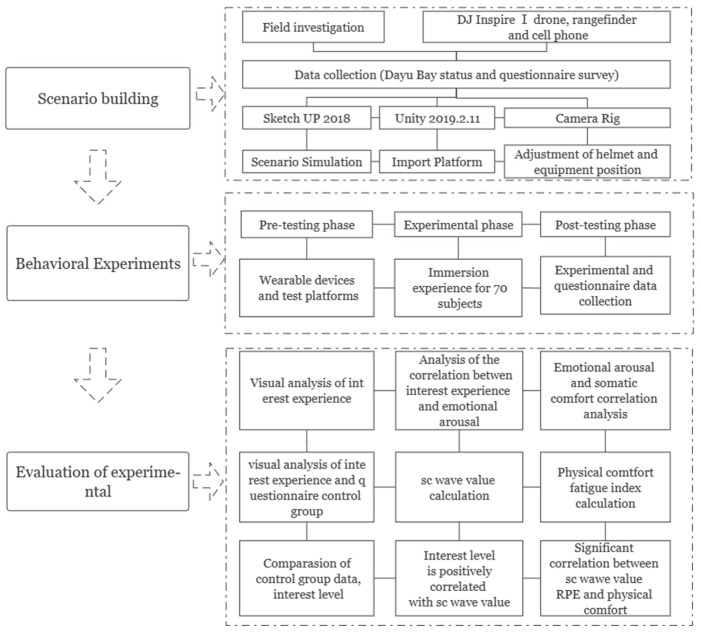
Mind map of innovation model. Source: Process on software production.

According to the logical relationship of research methods, this study forms an innovative model of immersive scenario simulation–somatic comfort assessment and climate change monitoring from the aspects of scene construction, behavioral experimentation and somatic comfort evaluation, as shown in Figure 7.

## 4. Research Results

Based on the statistical analysis of the pairwise correlation between the immersive experience and interest, interest and emotional arousal, emotional arousal and the somatosensory comfort level, the following results are obtained:

### 4.1. Subjects Showed the Highest Interest in the Ancient Tree Ecological Area after the Digital Roaming Landscape Immersive Experience

**Figure 8:**
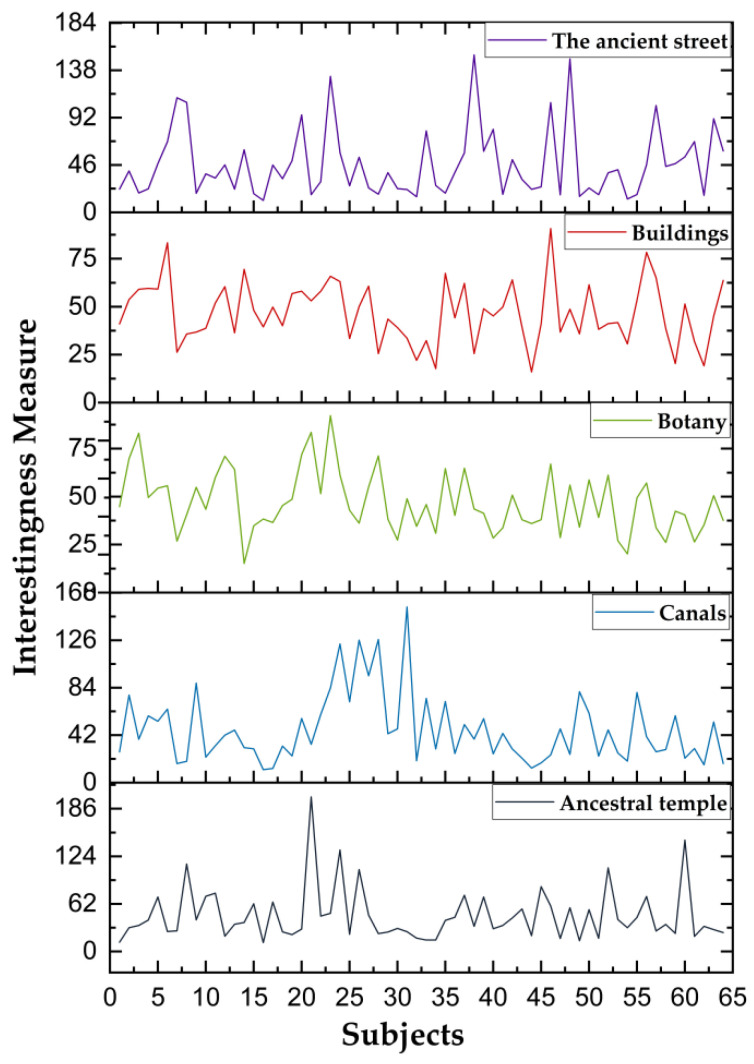
Trend graphs of interest level of 64 subjects in five interest areas. Source: The ErgoLAB platform was used to output the data, the *X-*axis represents subjects, and the *Y*-axis represents interest trends.

The interest level of the subjects’ somatosensory comfort experience in the digital landscape roaming scene as obtained. According to Equations (1)–(3), the trend in the interest level of 64 subjects was calculated and shown in Figure 8.

As shown in Figure 8, when the interest degree of 64 subjects was ≥1.13, the subjects had a high interest in the digital landscape roaming scene, which is consistent with Ward’s (2003) [46] research results. In addition, the interest degree of 64 subjects was higher than 1.13, with the maximum value of 7.73 and the minimum value of 2.82, which confirmed that the subjects were highly interested in the digital landscape roaming scene. According to the calculation results of Formula (3), the maximum interest of the subjects in the construction of plant landscape nodes and architectural landscape nodes was 9.22 and 9.02, and the minimum interest was 1.52 and 1.60. The maximum value of the degree of interest in ditch landscape node construction was 15.53, and the minimum value was 1.15. The maximum value of interest in the construction of street landscape nodes and ancient tree ecological area landscape nodes was 15.29 and 20.14, and the minimum value was 1.15, as shown in Table 2.

**Figure 9:**
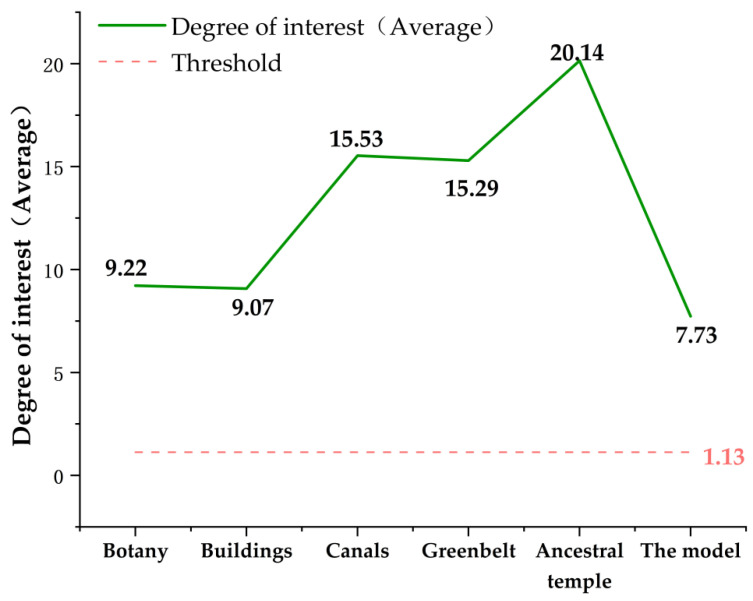
Comparison chart of interest trend. Source: Origin 2021 software production. The weight threshold of the interest degree was 1.13, and the maximum value of the interest degree was used to confirm that the subjects were most interested in the ancestral temple in the comparative analysis of the trend of interest degree changes.

As shown in Table 2, the order of the degree of interest in the interest area is ancient tree ecological area landscape node > ditch landscape node > street landscape node > building landscape node > vegetation landscape node. The statistical results of the experimental questionnaire showed that the subjects’ somatosensory comfort level in the roaming scene was ranked as ancient tree ecological area landscape node > architectural landscape node > street landscape node > canal landscape node > vegetation landscape node; this was consistent with the experimental results, as shown in Figure 9.

### 4.2. Subjects’ High Interest after Immersive Experience Is Significantly Correlated with Emotional Arousal

**Figure 10:**
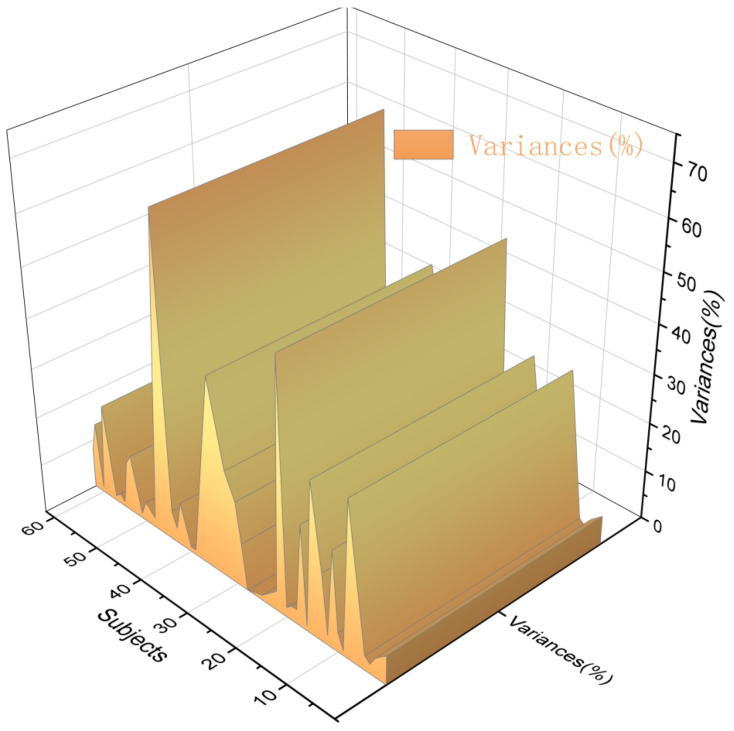
Trend chart of SC fluctuation variance. Source: Produced using origin 2021 software. SC fluctuation values of 64 subjects were calculated by SC variance values.

In this study, the variance in SC fluctuation in the electrodermal experiment was analyzed visually. The results showed that the mean SC variance of 64 subjects was 13.23%, of which 50 had SC values < 13%, accounting for 78.12% of the total number. The SC value of 45 students was less than 7%, accounting for 70.31% of the total number. Only 14 patients had an SC value ≥ 13%, accounting for 21.87% of the total population. This shows that 64 subjects had a low overall stress level and low cognitive load, as shown in Figure 10.

In order to investigate whether the interest experience of the subjects in the digital landscape roaming scene is related to positive emotions, a correlation analysis between the five groups of interest areas and SC was conducted, as shown in Table 3.

According to the analysis results in Table 3, SC was positively correlated with vegetation landscape nodes (r = 0.870, *p* = 0.00), architectural landscape nodes (r = 0.924, *p* = 0.00), canal landscape nodes (r = 0.261, *p* = 0.037) and ancient tree ecological area landscape nodes (r = 0.663, *p* = 0.00). There was no significant correlation between SC and street view nodes (r = 0.208, *p* = 0.099). It was confirmed that the variance value of SC fluctuation was low, that the degree of interest was high in the five types of digital landscape, and that the degree of interest was significantly correlated with positive emotional arousal.

### 4.3. The Subjects’ Low Emotional Arousal, Low Fatigue Level and High Somatosensory Sense Comfort Were Significantly Correlated

**Figure 11:**
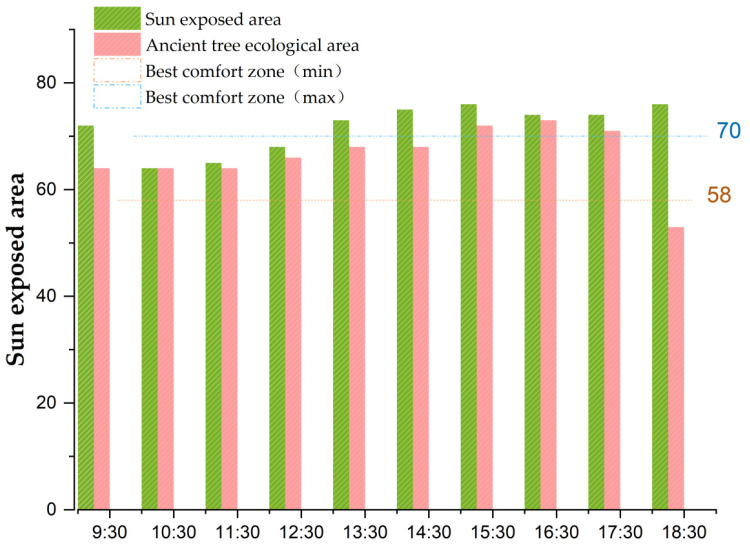
Numerical trend of somatosensory sense and comfort. Source: Origin 2021 software production. 58–70 is the best comfort zone, 50–80 is the comfort zone.

According to Formula (4), the results show that there were three periods in the optimal comfort range of 58–70 and three periods in the high temperature range of 75–100 in the sunlight-exposed area. There were six periods in the 58–70 optimal comfort zone and four periods in the comfort zone, without any comfort zone. It was proved that the somatosensory comfort level of the ancient tree ecological area was significantly better than that of the sunlight-exposed area, as shown in Figure 11.

**Figure 12:**
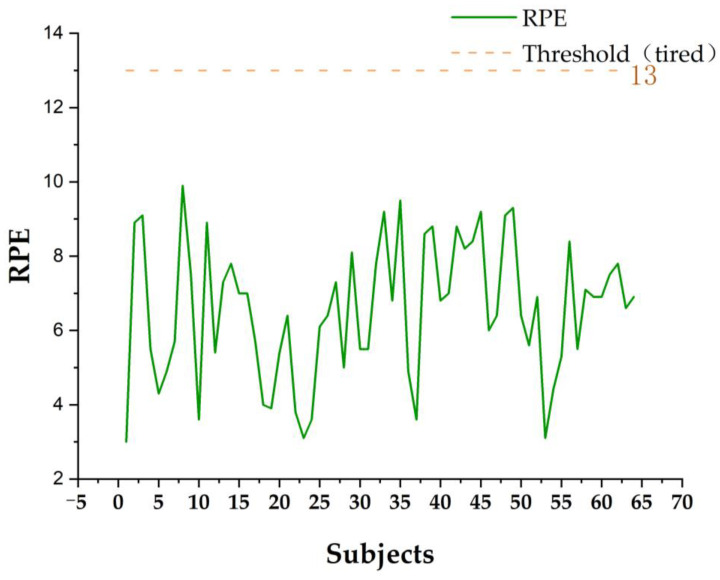
Numerical trend of RPE. Source: Origin 2021 software production. RPE 13 is the threshold, RPE ≥ 13 is the fatigue state.

According to the statistical results in Table 1, the RPE values of 64 subjects were all < 13 (57 of them were ≤9, accounting for 89.10% of the total number), and the heart rates were all lower than the 116.8–130.6 bpm range. It was confirmed that the subjects had no sense of fatigue and were at a relaxed and comfortable level in the digital landscape roaming scene, with a high somatosensory comfort level, as shown in Figure 12.

In this study, we conducted a correlation analysis between SC wave values, RPE values and the somatosensory comfort level; the results showed that SC wave values were significantly correlated with RPE values (r = 0.583, *p* = 0.00) and somatosensory comfort level (r = 0.257, *p* = 0.040). It was confirmed that emotional arousal is positively correlated with somatosensory comfort, and the smaller the SC wave value, the lower the RPE value, the smaller the HR fluctuation, and the higher the subjects’ sense of comfort; this shows the logical relationship between “interest experience–emotional arousal–body comfort level”, as shown in Table 4.

### 4.4. Somatosensory Comfort Can Effectively Distinguish the Optimal Comfort Level between the Ancient Tree Ecological Area and the Sunlight-Exposed Area

According to formula (4), the somatosensory comfort level of the ancient tree ecological area was in the best comfort range (58 ≤ *I_CHB_* ≤ 70), while the sunlight-exposed area was in the high temperature range (75 ≤ *I_CHB_* ≤ 100). The results showed that somatosensory comfort level could effectively distinguish the best comfort degree of the ancient tree ecological area and the sunlight-exposed area. The research results of Yau (2022) and Vanos (2012) also believe that somatosensory comfort level provides an important basis for extreme climate change monitoring [57,58]. Therefore, against the background of frequent global extreme weather events, this study was influenced by the subtle changes in the conductance of a human’s skin using sympathetic nerve monitoring technology, used ancient cultural heritage sites as the breakthrough point, monitored the somatosensory comfort level of extreme heat in an ancient ecological tree and sunlight-exposed area, and provided new ways to alleviate the problem of global extreme weather. As a result, the logical relationship of “SC fluctuation variance value–somatosensory comfort level–extreme climate change monitoring–global ecological and environmental governance” was formed.

## 5. Discussion

Finding 4.1 confirms that a higher gaze frequency and a longer gaze duration indicate that subjects are more interested in digital landscape roaming scenes, which is consistent with the findings of Chuang et al. (2015) and Machała (2020) [59,60]. Chuang and Machała propose an interactive information retrieval model for eye-tracking technology to analyze cognitive load. Based on this, this study further investigates subjects’ interest experience in digital landscape roaming scenarios. The change trend in the interest degree of the five groups of virtual landscape scene nodes confirms that the subjects had the highest interest degree in the ancient tree ecological area during the immersion experience, which is consistent with the research of Hessels et al. (2016) and Jongerius et al. (2021) [61,62]. Wang et al. analyzed the correlation between subjects’ interest in AOI. In this study, we not only use AOI to monitor the subjects’ interest level, but also analyze the reasons that this affects the subjects’ interest level. Moreover, the change trend in the maximum and minimum values of interest confirms that the subjects have a high interest in the immersive roaming experience, which is consistent with the research results of Fichtel et al. (2019) and Lorigo et al. (2008) [63,64].

Finding 4.2 confirms that the subjects’ interest was significantly correlated with emotional arousal, which was consistent with the results of Huang et al. (2022) [65]. After the immersion experience, the subjects used ancestral landscape, vegetation landscape and architectural landscape to conduct correlation analysis, which showed that the emotional arousal degree of the ancient tree ecological area and vegetation landscape node was lower; the SC wave value < 7% and the psychological cognitive load was small, showing a low-arousal emotional state. However, the architectural landscape nodes had a high emotional arousal; the SC wave value ≥7%, and there was a high psychological cognitive load, which is consistent with the research results of Lin et al. (2022) on SC wave value [66]. Lin et al. (2022) used SC wave value to analyze the healing effect of a digital landscape. According to AOI in digital landscape, this study analyzes the level of interest and the emotional arousal effect of the ancient tree ecological area and sunlight-exposed area. Through the tactile mental image theory, the results of the correlation analysis between the subjects’ interest and emotional arousal are consistent with experimental hypothesis 1. The reasons are analyzed as follows: the subjects’ interests is low, emotional arousal is high, and the psychological cognitive load is heavy. It is confirmed that interest is correlated with positive emotion, and the two are negatively correlated.

Finding 4.3 confirms that the high somatosensory comfort level of subjects was significantly correlated with low emotional arousal, which was consistent with the research results of Briefer et al. (2018) [67]. Briefer et al. believe that an appropriate temperature, humidity and wind speed give people a high somatosensory comfort level, and on this basis, this study further proposed that the high somatosensory comfort level of subjects is correlated with a low emotional arousal degree. According to Equations (4)-(6), when 6 ≤ RPE ≤ 12, the subject is in a state of low fatigue and low emotional arousal, and the SC wave value is < 7%, which confirms that the subject has a high somatosensory comfort level; this is consistent with the research results of Chen et al. (2022) [68]. When the temperature in the sunlight-exposed area is extremely high, and the somatosensory comfort level is ≥75 and the RPE index is ≥13, the subjects are in a state of high fatigue and high emotional arousal, which indicates that the subjects have a high psychological cognitive load and a low somatosensory comfort level; this is consistent with the research results of Kron (2013) [69]. He also believes that when the temperature is extremely high, the subjects’ sense of comfort will be reduced, that is, the degree of emotional arousal is low, and the subjects’ emotions fluctuate greatly. The results of the correlation analysis between the somatosensory comfort level and emotional arousal were consistent with experimental hypothesis 2.

Finding 4.4 confirms that somatosensory comfort level could effectively distinguish the comfort level between the ancient tree ecological area and the sunlight-exposed area, which is consistent with the research result of Li (2012) [70]. He believed that different plant communities would affect temperature, humidity and wind speed, and introduced discomfort level to evaluate the impact of different communities on somatosensory comfort level. This study takes ancient trees in cultural heritage sites as a starting point to monitor the somatosensory comfort level of ancient tree ecological areas and the sunlight-exposed area under an extreme heat environment. The logical relationship of “SC fluctuation variance value–somatosensory comfort level–extreme climate change monitoring–global ecological and environmental governance” was formed. The results are consistent with experimental hypothesis 3.

## 6. Conclusions

This study takes ancient trees as the entry point to monitor climate change through a behavioral preference experiment; it is a practical attempt at fulfilling the old Chinese saying “It is good to enjoy the shade under a big tree”, and makes it form a logical relationship between “behavioral preference, ancient tree ecology and climate change”. Dayu Bay is a national-level historical and cultural village and a typical historical and cultural heritage site. Its numerous ancient trees have become an important reference point for studying climate change. By comparing and analyzing the level of somatosensory comfort between the ancient tree ecological area and the sunlight-exposed area, it provides a new reference point for curbing global warming and extreme weather. It also reduces the negative perception of extreme weather through the construction of a body comfort model.

Somatosensory comfort level is a sensitive level, which has important value and practical significance for the objective evaluation of the human settlement environment. Ancient trees are one of the important measures that can be used to alleviate global heat load, and play a positive role in improving the environment, reducing energy consumption and the production of an urban heat island. The practical role of ancient trees in the environment is far more reliable and important than empty slogans such as global governance, ecological protection and environmental protection. Atake et al. (2007) propose that somatosensory comfort level is the direct perception of human body function to temperature changes. The skin and internal organs of the human body feel the changes in the internal and external environment and react to them through the nervous system [71]. Shi et al. (2012) believe that body comfort monitoring could obtain physiological indicators of human comfort under different climate conditions from the perspective of meteorology [72]. The temperature, wind speed and relative humidity of an area are used to monitor somatosensory sense and comfort, so that the monitoring results are more targeted. Rui (2019) takes ancient trees as the starting point to analyze the impact of plant communities on somatosensory comfort and environmental comfort, and under the frequent influence of extreme climate, monitors somatosensory comfort from ancient tree landscape nodes in cultural heritage sites; this provides new ideas for alleviating global warming and coordinating the harmonious development of human and nature [73]. Using a digital landscape scene to monitor body sensation and comfort can enhance the virtual immersive experience effect of subjects. Zhao (2017) believes that virtual landscape can provide subjects with a more objective immersive experience through the organic combination of VR, model design and ecological simulation [74]. Digital information, such as multimedia technology, virtual reality technology and display technology, can be used to restore and activate the sound and shape of cultural heritage, enhance people’s awareness of ecological protection, and guide global governance with the concept of the harmonious coexistence between human and nature [75].

This study also has certain limitations: Firstly, this study takes the proficiency of the handle operation as the control variable, which means that vertigo and movement sensitivity will affect the accuracy of the experimental data to some extent. Then, the pre-experiment can be used in the follow-up experiment, so that the subjects can perceive the experimental environment and handle operation mode in advance, so as to improve the accuracy of the experimental data. Secondly, during the experimental process, the amount of randomly invited subjects was small, and the overall sample size was still slightly insufficient. Finally, the method of studying the body comfort index through HR (heart rate) is affected by gender and age differences between men and women, so the experimental data will be biased to a certain extent. The next research will enhance the experience of body comfort by improving the accuracy of experimental data.

## Figures and Tables

**Table 1:** Related data of somatosensory sense and comfort.

	Ancient Tree Ecological Area			Sunlight-Exposed Area		
Time	*t* (°C)	*RH* (%)	*V* (m/s)	*t* (℃)	*RH* (%)	*V* (m/s)
9:30	26.1	84.50	17.50	29.2	56.30	18.30
10:30	27.2	82.30	18.60	29.1	60.40	20.80
11:30	27.1	75.40	17	30.2	55.20	17.50
12:30	28.3	70	13.20	31.1	57.20	15.20
13:30	28.3	68.10	9.80	32.4	60.10	8.70
14:30	29.3	57.20	8.60	33.5	60.20	7.30
15:30	30.3	59.50	5.20	33.2	60.10	6.50
16:30	30.2	57.40	4	33.1	65.30	10
17:30	29.1	63.20	6.30	32.2	70.40	9.20
18:30	27.4	92	8.50	31.1	75.20	5.60

**Table 2:** Statistics of maximum and minimum value of AOI.

AOI	Plant Landscape	Architecture Landscape	The Ditch Landscape	The Street Landscape	Landscape of Ancient Tree Ecological Area
The maximum	9.22	9.02	15.53	15.29	20.14
The minimum	1.52	1.60	1.15	1.15	1.15

**Table 3:** Correlative statistics of landscape node interest degree and emotion.

Interest in the Category	Sig	*p*	N
Plant landscape	0.00	<0.01	64
Landscape architecture	0.00	<0.01	64
Water landscape	0.037	<0.05	64
Landscape of ancient tree ecological area	0.00	<0.01	64
The street landscape	0.99	>0.05	64

**Table 4:** Correlation statistics of SC, RPE and *I_CHB_*.

		*RPE*	*I_CHB_*
**SC value**	The correlation coefficient	0.583 **	0.257 *
	*p* values	0.000	0.040

* *p* < 0.05 ** *p* < 0.01.

## Data Availability

Not applicable.
